# Detection of dengue virus type 2 of Indian origin in acute febrile patients in rural Kenya

**DOI:** 10.1371/journal.pntd.0008099

**Published:** 2020-03-03

**Authors:** Moses Muia Masika, Essi M. Korhonen, Teemu Smura, Ruut Uusitalo, Katariina Vapalahti, Dufton Mwaengo, Anne J. Jääskeläinen, Omu Anzala, Olli Vapalahti, Eili Huhtamo

**Affiliations:** 1 Department of Medical Microbiology, University of Nairobi, Nairobi, Kenya; 2 KAVI Institute of Clinical Research, University of Nairobi, Nairobi, Kenya; 3 Department of Virology, Medicum, University of Helsinki, Helsinki, Finland; 4 Department of Veterinary Biosciences, University of Helsinki, Helsinki, Finland; 5 Department of Geosciences and Geography, University of Helsinki, Helsinki, Finland; 6 Institute of Tropical and Infectious Diseases, University of Nairobi, Nairobi, Kenya; 7 Department of Virology and Immunology, University of Helsinki and Helsinki University Hospital, Helsinki, Finland; Universidade Federal de Minas Gerais, BRAZIL

## Abstract

Dengue virus (DENV) has caused recent outbreaks in coastal cities of Kenya, but the epidemiological situation in other areas of Kenya is largely unknown. We investigated the role of DENV infection as a cause of acute febrile disease in non-epidemic settings in rural and urban study areas in Kenya. Altogether, 560 patients were sampled in 2016–2017 in rural Taita–Taveta County (*n* = 327) and urban slums of Kibera, Nairobi (*n* = 233). The samples were studied for DENV IgM, IgG, NS1 antigen and flaviviral RNA. IgG seroprevalence was found to be higher in Taita–Taveta (14%) than in Nairobi (3%). Five Taita–Taveta patients were positive for flaviviral RNA, all identified as DENV-2, cosmopolitan genotype. Local transmission in Taita–Taveta was suspected in a patient without travel history. The sequence analysis suggested that DENV-2 strains circulating in coastal and southern Kenya likely arose from a single introduction from India. The molecular clock analyses dated the most recent ancestor to the Kenyan strains a year before the large 2013 outbreak in Mombasa. After this, the virus has been detected in Kilifi in 2014, from our patients in Taita–Taveta in 2016, and in an outbreak in Malindi in 2017. The results highlight that silent transmission occurs between epidemics and also affects rural areas. More information is needed to understand the local epidemiological characteristics and future risks of dengue in Kenya.

## Author summary

Dengue virus (DENV) is an emerging mosquito-borne global health threat in the tropics and subtropics. The majority of the world’s population live in areas at risk of dengue that can cause a wide variety of symptoms from febrile illness to haemorrhagic fever. Information of DENV in Africa is limited and fragmented. In Kenya, dengue is a recognized disease in coastal cities that have experienced recent outbreaks. We investigated the role of DENV infection as a cause of acute febrile disease in non-epidemic settings in rural and urban study areas in Kenya. We found DENV-2 in five febrile patients from rural Taita–Taveta, where no dengue has been reported before. Genetic analysis of the virus suggests it to be most likely of Indian origin. This Indian origin DENV-2 was detected in the Mombasa outbreak in 2013, in Kilifi in 2014, in Taita–Taveta in 2016 (our study samples) and again in the Malindi outbreak in 2017. The results suggest that dengue is unrecognized in rural Kenya and more studies are needed for local risk assessment. Our findings of virus transmission between epidemics contribute to better understanding of the epidemiological situation and origins of DENV in Kenya.

## Introduction

Family Flaviviridae, genus *Flavivirus*, includes important mosquito-borne human pathogens such as the yellow fever, Zika, West Nile and dengue viruses, all found in Africa [1]. Dengue viruses (DENV-1 to 4) have a significant impact on public health globally, but their impact in Africa is less characterized, and the available information and sequence data are fragmented [2, 3].

Serological data indicates the most active circulation of dengue in Kenya to occur in coastal region but low seroprevalences have also been reported from western and northern parts of the country [4–7].

First records of dengue in Kenya are from 1982, when there was a large outbreak caused by DENV-2 in the coastal area involving the cities of Kilifi and Malindi. The seroprevalence in the outbreak area was 52% after the outbreak [8]. In 2013 an outbreak caused by DENV-2 occurred in Mombasa. From this outbreak three DENV-2 isolates were obtained and sequenced [9], followed by ten strains of cosmopolitan genotype DENV-2 obtained from patient samples collected in 2014–2015 in Kilifi [10]. Again in 2017 an outbreak occurred in Malindi, where ten strains of related cosmopolitan genotype DENV-2 were isolated [11] demonstrating sustained DENV-2 circulation in Kenya.

The other serotypes of DENV have also been reported in Kenya, however the sequence data is very limited or lacking leaving the involved genotypes of the detected virus strains unknown. In 2011 an outbreak caused by DENV-3 was reported from Mandera region, north east Kenya [12]. During 2011–2014 samples of febrile patients were studied for dengue in Nairobi, northern and coastal Kenya resulting in detection of DENV-1–3, DENV-1 was reported to be the dominating serotype [13]. DENV-1 was also reported to be the dominating serotype in febrile children of Chuilambo and Kisumu, Western Kenya in 2014–2015, with all the four DENV serotypes detected by using DENV typing RT-PCR [14]. The main vector of DENV *Aedes (Stegomyia) aegypti* is known to be present in western parts and the coastal region of Kenya [15–18]. Sylvatic cycles of DENV are known to exist in Western Africa [19]. These have not been detected in Kenya, although sylvatic yellow fever virus is known to circulate and the environmental factors would likely allow sylvatic cycles of DENV as well [20].

Detection of dengue or other flaviviral infections requires the use of specific laboratory testing, as the symptoms are often unspecific. Except for malaria, diagnostic tests are usually not available at the point of care, especially in rural areas of Kenya [21]. The aim of this study was to determine the role of DENV infection (and potentially other flaviviruses) in acute febrile patients in a non-outbreak situation, in urban and rural areas of Kenya. We investigated the role of DENV infection as a cause of acute febrile disease in non-epidemic settings in rural and urban study areas in Kenya with molecular methods. In addition, we aimed to obtain information on the rate of past exposure to flaviviruses in these cohorts with serological methods.

## Methods

### Study design

The study sample collections were carried out in Kibera slum in Nairobi city and in rural Taita–Taveta County. Kibera slum is characterized by poor drainage, stagnant pools of water, congested and poorly litter, semi-permanent houses and a high human population density [22]. The rural Taita Hills area in Taita–Taveta County includes landscapes differing in altitude, vegetation and climate stretching over lowland savannah, Afromontane forested highlands and two national parks [23]. Taita–Taveta is traversed by new railway and road constructions and is proximal to the Kenyan coastline, which is known to have the highest prevalence of arboviral infections in Kenya [24].

**Fig 1:**
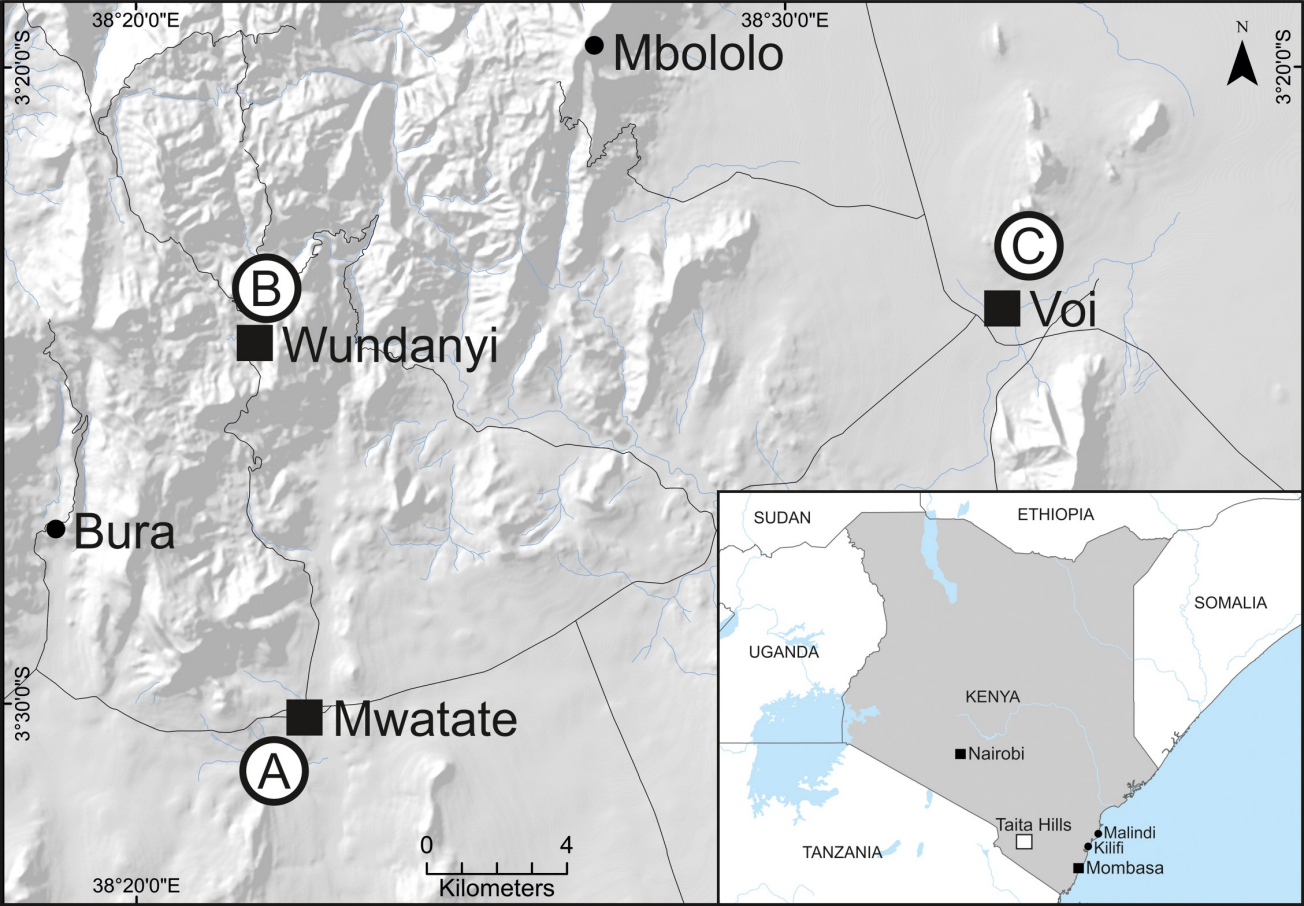
Maps of study areas in Kenya. The smaller map shows the locations of the study sites in Nairobi and the Taita Hills, as well as the dengue endemic coastal cities with reported dengue virus (DENV) -2 outbreaks (Mombasa 2013 and Malindi 2017) [11, 15]) and the town of Kilifi where DENV-2 was detected in febrile patients in 2014 [10]. The larger map shows more detailed sample collection locations in Voi, Wundanyi and Mwatate in Taita–Taveta County. The towns are designated with a square and villages with a circle. The hospitals where the samples were collected are designated with white circles and represent A) Mwatate Sub-County Hospital, B) Wundanyi Sub-County Hospital and C) Moi County Referral Hospital, Voi.

With approval from Kenyatta National Hospital-University of Nairobi Ethics and Research Committee (permit number P707/11/2015) samples were collected from voluntary febrile patients in six health facilities in Taita–Taveta in April to August 2016 and Kibera in February to June 2017 (Fig 1). Adult subjects provided written informed consent, and a parent or guardian of any child participant provided written informed consent on the child’s behalf. Any patient with a temperature of 37.5°C or higher was eligible for inclusion in the study. A total of 560 samples (sera or plasma) were collected in this study: 327 from Taita–Taveta County and 233 from Kibera slum in Nairobi. The samples were taken from the patients when they first entered the healthcare facility and represent the acute phase of febrile illness ranging from 1–14 days since onset of fever (median = 2 days, interquartile range = 2 days) (Table 1). Sociodemographic and clinical data were collected from all consenting patients using a questionnaire administered by a clinician. Serum samples were collected from all study participants in Taita–Taveta (*n* = 327) and plasma samples from the participants in Kibera (*n* = 233). The samples were initially stored at -20°C in the Taita—Taveta area (for up to three weeks before getting them to Nairobi) prior to storage at -80°C.

**Table 1:** Demography, exposures and symptoms in the total study population (*n* = 560).

	Taita- Taveta		Nairobi		Other*	
**Variable**	n	%	n	%	n	%
No. of subjects	322	57.5	230	41.1	8	1.4
‘dengue marker positive’	47	14.6	8	3.5		
	**<30**		**≥30**		**Missing**	
	n	%	n	%	n	%
Age	365	65.18	184	32.86	11	1.96
	**Female**		**Male**		**Missing**	
	n	%	n	%	n	%
Gender	287	51.25	263	46.96	10	1.79
**Patient history**	**Yes**		**No**		**Missing**	
	**n**	**%**	**n**	**%**	**n**	**%**
Travel history	142	25.4	413	73.8	5	0.9
HIV positive	77	13.8	483	86.3	0	0.0
Malaria positive	59	10.5	501	89.5	0	0.0
Contact with goats	160	28.6	400	71.4	0	0.0
Contact with cattle	123	22.0	437	78.0	0	0.0
Contact with sheep	38	6.8	522	93.2	0	0.0
Contact with swine	1	0.2	559	99.8	0	0.0
Contact with chicken	236	42.1	324	57.9	0	0.0
Contact with dogs	95	17.0	465	83.0	0	0.0
Contact with cats	151	27.0	409	73.0	0	0.0
Contact with rodents	345	61.6	215	38.4	0	0.0
Contact with bats	100	17.9	460	82.1	0	0.0
Rash	34	6.1	526	93.9	0	0.0
Joint pain	209	37.3	351	62.7	0	0.0
Myalgia	190	33.9	370	66.1	0	0.0
Vomiting	122	21.8	438	78.2	0	0.0
Diarrhoea	77	13.8	483	86.3	0	0.0
Bleeding tendency	2	0.4	558	99.6	0	0.0
Headache	162	28.9	398	71.1	0	0.0
Sore throat	39	7.0	521	93.0	0	0.0
Cough	141	25.2	419	74.8	0	0.0
	**Mean**	**Std**				
Temperature	38.61	0.63				

*Place of residence not known.

### Antibody and antigen testing

**Fig 2:**
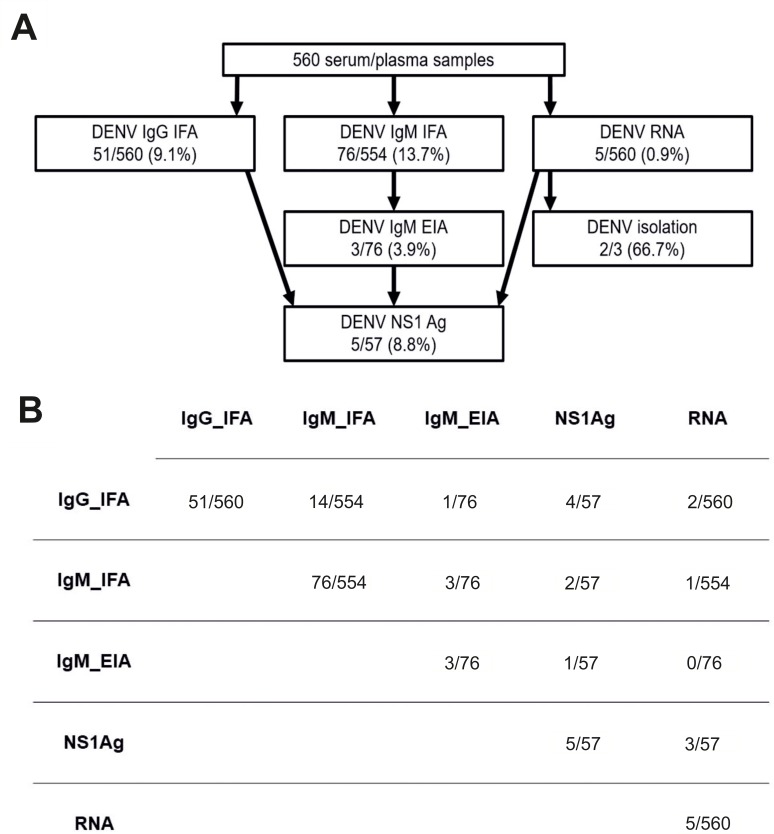
Diagnostic assays and results: a) workflow and results for flaviviral diagnostic tests; b) dengue serological assays and RT-PCR results.

Initial IgG and IgM antibody screening was done using an in-house immunofluorescence assay (IFA) with DENV-3 (H87) antigen [25–27]. The samples were diluted 1:20 in phosphate-buffered saline for testing in IFA. Samples that were found to be positive for IgG at 1:20 dilution were further titrated until the last positive dilution was reached. The IgG-positive samples were treated with GullSORB IgG inactivation reagent (Meridian Bioscience, Cincinnati, USA) prior to IgM IFA testing. Known DENV IgG and IgM positive samples were used as positive controls in the IFA tests. For the samples reactive by IgM IFA, a commercial dengue IgM enzyme immunoassay (EIA) was performed according to the manufacturer’s instructions (Capture DXSelect, Focus Diagnostics, Cypress, USA). Samples that were found to be positive in DENV IgM EIA, IgG IFA or viral RNA screening were additionally tested for dengue NS1 antigen using Platelia Dengue NS1 Ag test (BIORAD). If there was enough sample volume left, DENV IgG IFA positive samples were further tested for anti-Zika (ZIKV) virus IgG using a commercial ELISA test (Euroimmun, Luebeck, Germany), and samples positive for DENV IgM IFA were tested for anti-ZIKV IgM using an ELISA test (Zika IgA/M, Euroimmun, Luebeck, Germany) according to the manufacturer’s instructions (Fig 2A).

### Nucleic acid extraction and flaviviral RNA detection

Nucleic acids were extracted from 200μl of serum or plasma sample using a MagNa Pure LC instrument and MagNa Pure LC Total Nucleic Acid Kit (Roche, Basel, Switzerland) with an elution volume of 50μl. A pan-flavivirus RNA screening, targeting the NS5 gene, was performed using a real-time SYBR green one-step RT-PCR assay followed by a semi-nested amplification and agarose gel electrophoresis as described in detail by Moureau et al. [28, 29]. A total of 5μl of the nucleic acid extract was used as a starting material according to the Moureau et al. protocol, with the modification of using DreamTaq DNA polymerase (Thermo Fisher Scientific) in the semi-nested amplification step. Tick-borne encephalitis virus strain Kumlinge A52 (a non-endemic flavivirus to Kenya) RNA was used as a positive control in the pan-flavivirus RNA screening.

Additional DENV-2 envelope (E) gene region nested RT-PCR was done for selected samples. The RT step was done using Maxima reverse transcriptase, random hexamers and RiboLock RNase inhibitor following the manufacturer’s instructions. The PCR steps were done using DreamTaq DNA polymerase (Thermo Fisher Scientific, Waltham, USA) using DENV-2 E-gene specific primers: first round forward primer 5′-CATGGATGTCATCAGAAGGG-3′, first round reverse primer 5′-CCTTTRATGTCTCCT GTCAT-3′, second round forward primer 5′-GGRTYYTGAGACATCCAGG-3′ and second round reverse primer 5′-TCTGRTGTTATYTGTTTCCAC-3′.

The first and second round PCRs were carried out in 50μl reaction volumes using 2μl of cDNA in the first round PCR. A total of 2μl of the first round PCR product was used as a template in the second round PCR. The thermal cycling conditions (for both rounds) included initial denaturation at 95°C for 3min followed by 35 times a cycle at 94°C for 40s, 50°C for 50s, and 72°C for 1.5min.

### Virus isolation

Virus isolation was attempted from DENV-2 RNA positive samples where a suitable amount of material was available (*n* = 3) in Vero E6 (ATCC CRL-1586) and C6/36 *Aedes albopictus* cells (ATCC CRL-1660) as described previously [30]. In brief 50μl of sera was inoculated to cells grown in 25cm^2^ culture bottles.

### Sequencing

The RT-PCR products of pan-flavivirus RNA screening (~150 bp) and DENV-2 E-gene RT-PCR (~1500 bp) were Sanger sequenced (DNA sequencing and genomics laboratory, Institute of Biotechnology, University of Helsinki). For obtaining complete genome data, next generation sequencing (NGS) was attempted from RNA extracted from patient samples or virus isolation samples using Illumina MiSeq (Illumina, San Diego, USA). The samples were prepared using a protocol modified from Conceicao-Neto et al. [31]. Briefly, the samples were filtered (0.4 μm filters), nuclease digested with benzonase (Millipore, Burlington, USA) and micrococcal nuclease (New England Biolabs, Ispwich, USA), and finally precipitated with polyethyl glycol (Abcam, PEG virus precipitation kit). Nucleic acid extraction was conducted using a QIAamp Viral RNA Mini Kit (Qiagen, Hilden, Germany), followed by reverse transcription and amplification using a WTA2 Whole Transcriptome Amplification Kit (Sigma Aldrich, Saint Louis, USA). The amplified DNA was purified using a GeneJet PCR Purification Kit (Life Technologies, Carlsbad, USA) and libraries prepared using a Nextera XT kit (Illumina, San Diego, USA) according to the manufacturer’s instructions. The library fragment size was estimated using agarose gel electrophoresis and the concentration measured using a Qubit Broad-Range dsDNA Assay Kit (Life Technologies, Carlsbad, USA) and NEBNext Library Quant kit (New England BioLabs). The samples were sequenced using a MiSeq V2 reagent kit with 150 bp reads.

### Sequence analysis

The initial virus species designation for the sequences obtained from screening RT-PCR product by Sanger sequencing was conducted using a basic local alignment search tool (BLAST). Raw NGS sequence reads were trimmed and low quality (quality score <30) and short (<50 nt) sequences removed using Trimmomatic [32]. The trimmed sequences were *de-novo* assembled using a MIRA assembler (version 4.9.5. http://mira-assembler.sourceforge.net) [33] followed by reassembly against the *de-novo* assembled consensus sequences using the BWA-MEM algorithm implemented in SAMTools version 1 [34, 35]. The mean coverages were: 6710 (range: 424–15 298) for strain 222_Voi_Kenya; 1679 (range 410–5397) for strain c96_Wundanyi_Kenya and 51 (range 8–163) for strain 85A_Wundanyi_Kenya.

Complete coding sequences of the DENV-2 cosmopolitan genotype were retrieved from the GenBank (accessed in 1 March 2019) for phylogenetic analysis. Representative strains of the other DENV-2 genotypes (American, Asian American, Asian I, Asian II and sylvatic) were also included in the initial analysis. The initial analysis suggested that the Kenyan DENV-2 strains form a distinct cluster together with strains of Indian origin. These strains were selected for a more detailed analysis. Another data set was constructed of the E-gene that contained all available E sequences that clustered together with the Kenyan/Indian subcluster identified on the basis of initial complete coding sequence analysis.

The sequences were aligned using MUSCLE [36] and the substitution model was estimated using bModelTest [37] implemented in BEAST2.5 software [38]. The phylogenetic trees were constructed using the Bayesian Monte Carlo Markov Chain (MCMC) method implemented in BEAST version 1.8.0 [39] using the TN93 model of substitution with 4-category gamma-distributed variation among sites and a proportion of invariant sites. Three molecular clock models (strict, log-normal relaxed and exponential) and three demographic models (constant, exponential and Bayesian skyline) were compared by estimating marginal likelihoods by stepping stone and path sampling methods [40, 41]. Bayes factors (BFs) were calculated for each pair of models. Both stepping stone and path sampling methods supported a log-normal relaxed clock and an exponential growth demographic model over the other models (BF >60). The analyses were run for 50 million states and sampled every 5000 steps. Posterior probabilities were calculated with a burn-in of 10% and checked for convergence using Tracer version 1.7 [39]. The analyses were carried out on the CSC server (IT Center for Science, Espoo, Finland).

### Statistical tests of questionnaire data

Statistical analyses combining diagnostic test results and questionnaire data were performed for 499 patients out of the total 560 patients (61 patients were excluded because positive IgM IFA could not be confirmed by IgM EIA or due to incomplete questionnaire data). We used SAS 9.4 (SAS Institute, Cary, NC, USA) in the analyses, which included Fisher’s exact test and logistic regression analyses with the level of significance set at 95% (*p*-value <0.05). Multivariable logistic regression analysis was used to find the most significant risk factors and to control the effects of confounding variables. Based on diagnostic assay results, patients were divided into groups of ‘DENV marker positive’ (positive in any test) and ‘DENV marker negative’ (negative in all tests).

This status (DENV marker positive or negative) served as a response variable, and age, gender and county were defined as confounding variables. Continuous age was reduced to two categories with age <30, or **≥**30. First, potential risk variables were individually examined in the model with confounding variables. Then, the variables that proved to be significant in these models were tested in different combinations in a model with confounding variables to find the best multivariable model with the most significant risk factors. Interactions and multicollinearity between all variables in the models were tested. The phi coefficient was utilized in measuring multicollinearity and a value of >0.5 was set as a limit for strong multicollinearity [42]. The best model was selected according to the Pearson goodness-of-fit, AIC and AUC values of the model.

## Results

### Patient characteristics and associations with DENV marker positivity

A total of 560 samples (sera or plasma) were collected in this study; 327 from Taita–Taveta County and 233 from Kibera slum in Nairobi (Table 1). The samples were taken from the patients when they first entered the health care facility, and represent the acute phase of febrile illness ranging from 1–14 days since onset of fever (median = 2 days, interquartile range = 2 days) (Table 1). The patients included 322 residents of Taita–Taveta County and 230 of Kibera, Nairobi. The place of residence was not known for eight patients. A quarter of all patients had travelled outside their county of residence within the previous month. No trips abroad were reported by any of the patients. In addition, patients also reported joint pain (37%), myalgia (34%), headache (30%), cough (25%), vomiting (22%), diarrhoea (14%), sore throat (7%) and rash (6%). Information of HIV positivity was available for 14% and malaria test was positive in 11% of all the study patients (Table 1).

According to Fisher’s exact test (S1 Table), DENV marker positivity was significantly more common in Taita-Taveta compared with Nairobi, in age group ≥30 years compared with age group <30 years and among females compared with males. In addition, contact with goats, chickens or bats was more common among ‘DENV marker positive’ than ‘DENV marker negative’ patients. When studied separately in Taita–Taveta patients, similar significant differences were found in age, gender and travel history (S1 Table). Further statistical testing included examining the effects of risk factors in multivariable models which included confounding factors. In the county of Taita-Taveta only, the best model included the variables age, gender and travel history. In the whole study group of Taita-Taveta and Nairobi together, also significant interaction between the variables chicken and age was found (Table 2).

**Table 2:** Best logistic regression models for DENV in the whole study group (Taita Taveta and Nairobi combined) and in Taita Taveta solely.

		Model fit statistics							
	Variables	AIC	AUC	Pearson	p	OR	Lower 95%CI	Higher 95%CI	
**Whole study group**	Age_group	287.52	0.80	0.99	< .0001	.	.	.	
	Gender: female vs male				0.02	2.32	1.19	4.71	
	County*TravelHistory				0.23	5.2	2.24	11.94	^1)^
						1.71	0.35	8.30	^2)^
	Age_group*Chicken				0.01	0.5	0.22	1.19	^3)^
						2.74	0.95	7.95	^4)^
**Taita Taveta**	Age_group: > = 30 vs <30	229.85	0.74	0.91	0.00	3.46	1.67	7.60	
	Gender: female vs male				0.02	2.51	1.21	5.51	
	Travelhistory				< .0001	5.82	2.53	13.69	

^1)^ Travel history at County = Taita Taveta

^2)^ Travel history at County = Nairobi

^3)^ Chicken*Age = > 30 yrs

^4)^ Chicken*Age < 30 yrs

Indicators of model goodness: AIC, AUC and Pearson Goodness-of-Fit.

No significant multicollinearity was found between the variables in the models. Both in the whole study group and Taita-Taveta solely, the confounding factors turned out to be risk factors as well. The low number of ‘DENV marker positive’ patients (*n* = 8) did not allow a more detailed analysis of the patients sampled in Nairobi.

### Diagnostic assay results

The IgG seroprevalences of patients sampled in Taita–Taveta was higher (14%) than in patients sampled in Nairobi (3%). Altogether, 9.1% (51/560) of samples were found to be positive in DENV IgG IFA and 3.9% (3/76) of IgM IFA positive samples were also positive by commercial IgM EIA (Fig 2B). The IgG IFA titres ranged from 10 to 2560. 12 samples had low titres (10–20), 31 samples had intermediate titers (40–160) and 7 samples had high titers (640–2560). For one sample, the titre could not be determined after the initial screening dilution due to a lack of sample volume. In ZIKV antibody tests, 1/51 and 1/57 were found to be positive for ZIKV IgG and IgM, respectively.

Acute DENV infection was documented by molecular methods in five patients who were found to be positive in pan-flavivirus RNA screening (0.9%) and further confirmed by sequencing. All these patients were sampled in Taita–Taveta County (Table 3). Four of the five PCR-positive samples were collected in May 2016 and one in June 2016. The DENV NS1 antigen assay was positive in three of the viral RNA positive patients. Virus isolation was attempted for three DENV-2 RNA positive samples, for which sufficient amounts of sample were available. The virus was successfully isolated from two samples in C6/36 cells (Table 3, Fig 2A) Both of the strains showed cytopathic effects in infected cells (as rounding and appearance of loose cells) after subcultures on days 20 and 24.

**Table 3:** Characteristics of dengue virus RNA positive patients.

Sample ID	Collection place and date	Travel history	Symptoms (days)	Patient age/ sex*	Sampling day**	IgG (IFA) titre	IgM (IFA)	IgM EIA dengue	Pan- flavi virus RT-PCR	DENV NS1 Ag	Virus isolation	Sequence confirmation
**76**	Wundanyi 16-May-2016	Nairobi	Fever (2)	3 F	2	<20	<20	Neg	Pos	ND	ND	DENV-2, partial cds NS5 132 bp (Sanger) ***
**85A**	Wundanyi 10-May-2016	Mombasa	Fever (2) Joint pain (2) Myalgia (2)	16 F	2	640	20	Neg	Pos	Pos	Pos	DENV-2, complete cds (NGS from virus isolate) GenBank accession: MK473385
**c96**	Wundanyi 4-May-2016	Mombasa	Fever (2) Joint pain (2) Myalgia (2) Headache (2)	19 F	2	<20	<20	Neg	Pos	Pos	Pos	DENV-2 complete cds (NGS from virus isolate) GenBank accession: MK473384
**509**	Mwatate 24-Jun-2016	No recent travelling	Fever (3) Myalgia (3) Rash (3)	46 M	3	<20	<20	Neg	Pos	Neg	Neg	DENV-2, partial cds, E-gene, 1485 bp Sanger GenBank accession: MK473383
**222**	Voi 4-May-2016	Mombasa	Fever (2)	17 M	2	40	<20	Neg	Pos	Pos	ND	DENV-2, complete cds. direct NGS from sample, GenBank accession: MK473386

* M = male, F = female

** sampling day since onset of fever *** available from the authors upon request; DENV-2 = Dengue virus type 2

In addition to these five confirmed dengue patients, acute flaviviral infection was suspected in three additional patients (0.5%) with a positive IgM EIA (Capture DXSelect, Focus Diagnostics) result. The patients positive for DENV IgG only (6.4%) were considered to have old flaviviral immunity (Fig 2B).

### Sequencing and phylogenetic analysis

Using Sanger sequencing, the pan-flavivirus screening RT-PCR products (*n* = 5, ~ 150bp) were identified as DENV-2 with high identity matches in the BLAST search. A total of three complete DENV-2 coding sequences and one E-gene sequence were obtained in this study (Table 3). The attempt to obtain longer viral sequences for analysis were not successful in one sample (number 76). This sequence was 99–100% identical to the other sequences obtained in this study.

**Fig 3:**
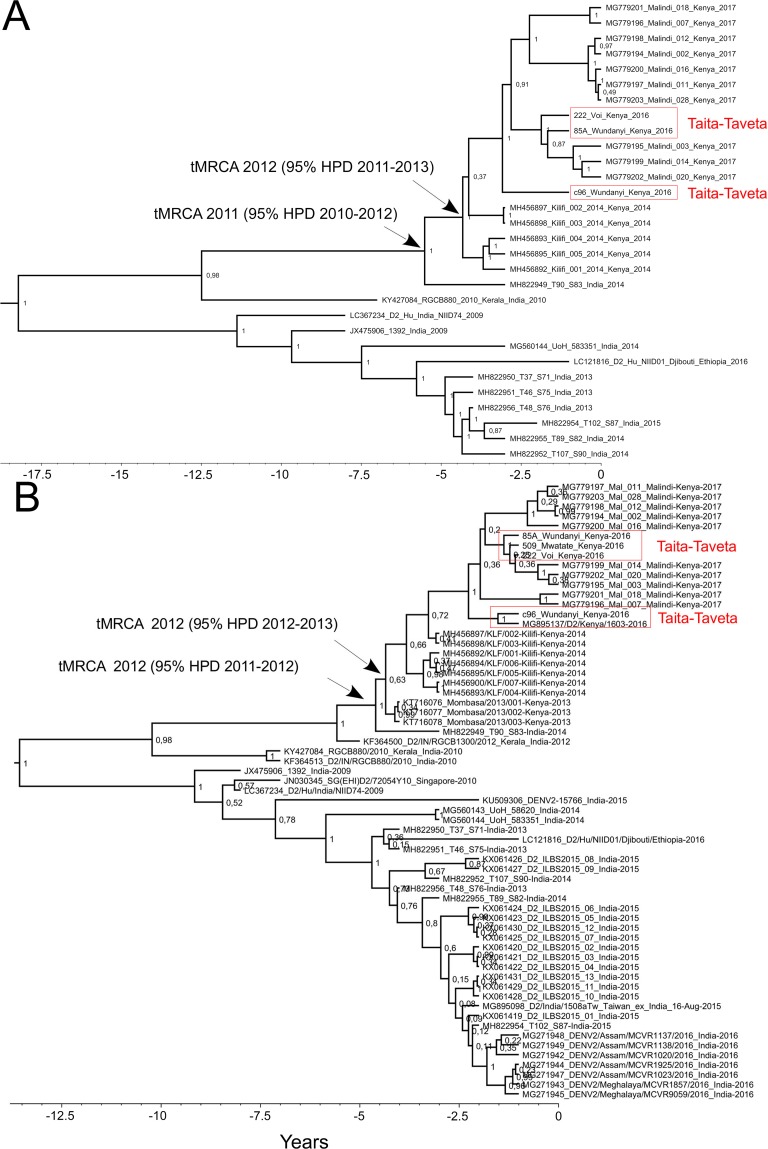
Phylogenetic trees of dengue virus 2 a) Complete open reading frame sequences and b) envelope gene sequences. In a) the nodes of respective tMRCAs are shown with arrows. For both a) and b), maximum clade credibility tree of dengue virus 2 are presented, including strains that had >98% sequence identity in the BLAST search. Trees were constructed using the Bayesian MCMC method with the TN93 model of substitution with a 4-category gamma-distributed variation among sites and a proportion of invariable sites, log-normal relaxed clock model and exponential growth demographic model. Posterior probabilities are shown for each node.

Phylogenetic analysis based on all the available DENV-2 cosmopolitan genotype complete coding sequences (*n* = 30) suggested that the Kenyan strains form a single monophyletic cluster, and the strain T90_S83 from New Delhi, India (year 2014) formed an out-group for all the recent Kenyan DENV-2 strains. These strains clustered further with strain RGCB880 from Kerala, India (year 2010) (Fig 3A).

Since there are more E-genes than complete genomes in the GenBank, a distinct data set was constructed from the closest BLAST search matches to the Kenyan DENV-2 E-genes. The E-gene sequence analysis (1485 bp) included 63 sequences from years 2009–2017 (Fig 3B). Consistently with the complete coding sequence analysis, all the Kenyan sequences formed a monophyletic group most closely related to sequences from New Delhi, India, in the year 2014 [43] (tMRCA: 2012 [95% HPD 2011–2012]). In addition to the strains mentioned above, the sister group included sequences originating from India and Singapore. Interestingly, the strain from a traveller patient returning from a trip to Djibouti and Ethiopia in 2016 was found to be closely associated with the New Delhi strains in the E-gene analysis. The recent (years 2016–2017) Kenyan strains formed a monophyletic cluster that was further divided into four highly supported subclusters. Strain c96 from Wundanyi in 2016 clustered together with a strain isolated from a patient travelling from Kenya to Taiwan in March 2016, whereas the strains 509 from Mwatate, 85A from Wundanyi and 222 from Voi in 2016 clustered together with three strains from Malindi in 2017.

The molecular clock analysis dated the most recent common ancestor (MRCA) for the Kenyan strains in year 2012 (95% HPD 2012–2013); a year prior to the 2013 epidemic in Mombasa. For the sequences sampled in this study and those sequenced from Malindi, the time to the most recent common (tMRCA) was 2014 (95% HPD 2014–2015).

## Discussion

Neither of our study areas had documented previous DENV outbreaks. Dengue outbreaks have occurred previously mainly in the coastal area of Kenya, and although some cases have been detected in Nairobi, it is not considered dengue endemic area with sustained transmission. The 3% seroprevalence of IgG against flaviviruses in Kiberan febrile patients was in line with the previous general population survey of Nairobi, where IgG seroprevalence was 3.5% in 2007 [24]. No signs of acute flavivirus/dengue infections were detected in any of the Kiberan patients.

No previous information was available for rural Taita–Taveta County regarding flavivirus seroprevalence or DENV transmission. The flavivirus IgG seroprevalence in Taita–Taveta was found to be 14%, which is considerably higher than in Nairobi but yet notably lower than in coastal region (34–67%) [4, 5]. The location of Taita-Taveta near to the dengue endemic coastal counties is likely a factor contributing to the higher seroprevalence [4, 24, 44]. The result may also be influenced by patient age, as the adult patients sampled in Taita–Taveta were probably more likely to be exposed to DENV or other flaviviruses during their life, than the younger patients sampled in Nairobi. The seroprevalence data obtained from this study cohort of febrile patients may not represent the general population in the region, and further studies with comparable study cohorts, such as general population would be needed for evaluating the regional exposure to flaviviruses in different regions of Kenya.

The lack of convalescent samples limited the further testing and interpretation of the serological results obtained from single time point samples. The IgG positivity alone without other positive test results was considered old immunity, however this may be due to other past flavivirus exposure than DENV. The serological cross-reactions are common between different flaviviruses, and Kenya is known to be endemic, in addition to DENV, to West Nile virus, yellow fever virus (YFV) and Wesselsbron virus [5]. YFV vaccination status of the patients was not known, but no routine vaccinations were ongoing in the study areas. As the study was targeted at acute phase febrile patients and the sample volumes were limited, the typing of IgG antibody specificities via neutralization assays was not attempted. Notably, only two DENV seropositive patients were found to be positive also for ZIKV NS1 specific antibodies: one for IgG and one for IgM. Both of these patients reported recent travel to western Kenya where ZIKV neutralizing antibodies have been previously detected [7]. The reason for the high number of patients positive for DENV IgM IFA but negative in all other tests (*n* = 59) remains unclear and raises questions of the reliability and specificity of this in-house test. However, taking into account the timing of sampling and dengue marker appearance in dengue patients [45], the used diagnostic methods should have detected acute DENV or other flaviviral infections.

The statistical analyses of questionnaire data was used to investigate associations of demographic data to diagnostic findings. The multivariable modelling of the available data obtained suggested that age (>30 years), female gender and travel (especially among the Taita–Taveta patient group) were significant risks factors for being positive in the flavivirus diagnostic tests. Notably, the travel history of the patients in this study included only domestic travel. Intriguingly, among younger patients, an association was found between contact with chicken and diagnostic test positivity. Whether this translates to environmental characteristics such as the presence of water containers that could influence vector breeding and abundance remains to be elucidated. However, this effect was not detected in patients over 30 years of age.

DENV-2 RNA was detected in five patients sampled in Taita–Taveta. The patients’ travel history implied that most of these infections could have originated in Mombasa (3/5) or Nairobi (1/5). One of the DENV-2 RNA positive patients was sampled in Mwatate and had not travelled in the preceding month. This would be compatible with local transmission of DENV-2 in the region. Interestingly, *Aedes* (*Stegomyia*) *aegypti*, which has been mainly associated with urban settlements in Kenya [16, 18], was repeatedly observed in some villages of Taita–Taveta County [46]. Our ongoing studies of the local mosquitoes may provide further evidence of the occurrence of the local transmission of DENV in Taita–Taveta.

The sequence analysis was restricted due to the limited amount of DENV sequence data obtained in this study, and available in public databases from previous studies in Kenya and the neighbouring countries. In this study, a single DENV serotype (DENV-2) and genotype (Cosmopolitan) was detected. Our sequence data from Taita–Taveta, located inland approximately 150 kilometres from the coast, is from the year 2016 and thus timed between the two large dengue outbreaks of the coastal cities of Mombasa in 2013 and Malindi in 2017. The findings are in line with previous work, which used complete genomic data and concluded that the 2017 Malindi outbreak strains were clonally derived and closely related to strains from India [11]. Our analysis further estimated the timing of the introduction of this lineage to Kenya a year before the 2013 Mombasa outbreak. The currently available data suggest, that after the 2013 Mombasa outbreak the virus spread towards the northeast along the coast. The virus was detected from febrile patients in Kilifi in 2014 [10] and later caused the Malindi 2017 outbreak [11] (Fig 1). In our analysis, the sister group to all the Kenyan cosmopolitan DENV-2 strains included Indian strains and interestingly also a strain originating from Djibouti or Ethiopia in 2016 from a traveller patient. This suggests that dispersal of DENV-2 between Africa and India has occurred more than once. Globalization has probably facilitated the introduction of the Indian DENV to Kenya, as Kenya along with other African countries interacts actively with India for trade and travel [47], and India is hyperendemic for DENV [48].

To conclude, we detected unnoticed DENV-2 transmission outside epidemics and previously known endemic regions in rural Kenya. Dengue was not a common cause of acute febrile disease in the study area of rural Taita-Taveta. However, as asymptomatic people were not included in this study, the true level of infections remains to be determined. The subclinical infections enable the silent transmission and may constitute the majority of the infections in an non-outbreak situation [49, 50]. The unrecognition of dengue due to lack of diagnostic testing is likely to lead to underestimates of its impact to the public health. Further studies would be needed for assessing the disease burden and for understanding the dynamics, maintenance and spread of dengue from the endemic coastal Kenya area to new areas. The monitoring of the population exposure would be needed in areas where the vector *Aedes* (*Stegomyia*) *aegypti* is present for risk assessment, preparedness and planning of possible prevention measures.

## Supporting information

S1 Table Frequencies and fisher's exact test p values of confounding and risk factors among “DENV marker positive” and “DENV marker negative” patients in whole study group and in Taita–Taveta solely. (DOCX)
