# Supplementary material for: Detection of dengue virus type 2 of Indian origin in acute febrile patients in rural Kenya
Source: PLoS Negl Trop Dis. 2020 Mar 3;14(3):e0008099. doi: 10.1371/journal.pntd.0008099 (PMC7069648; doi:10.1371/journal.pntd.0008099)
Supplement: S1 Table — (DOCX) [file pntd.0008099.s001.docx]

|  | | | | | | | | | | | | | | |
| --- | --- | --- | --- | --- | --- | --- | --- | --- | --- | --- | --- | --- | --- | --- |
| **Confounding factors** |  | **Whole study group** | | | |  |  |  | **Taita Taveta** | | |  |  |  |
|  | **Group** | **Positive** | | **Negative** | | **Missing** | **Fisher^**)^** |  | **Positive** | | **Negative** | | **Missing** | **Fisher^**)^** |
|  |  | n | % | n | % | n | p |  | n | % | n | % | n | p |
| Age_group | Age < 30 | 19 | 35 | 287 | 65 | 11 | **<.0001** |  | 15 | 32 | 150 | 59 | 6 | **0.00** |
|  | Age >=30 | 35 | 64 | 144 | 33 |  |  |  | 31 | 66 | 101 | 39 |  |  |
| Gender | Female | 37 | 67 | 217 | 49 | 9 | **0.02** |  | 33 | 70 | 127 | 50 | 7 | **0.02** |
|  | Male | 18 | 33 | 215 | 49 |  |  |  | 14 | 30 | 122 | 48 |  |  |
| County | Taita Taveta | 47 | 85 | 256 | 58 | 0 | **<.0001** |  |  |  |  |  |  |  |
|  | Nairobi | 8 | 15 | 185 | 42 |  |  |  |  |  |  |  |  |  |
| **Potential risk factors of DENV** | | |  |  |  |  |  |  |  |  |  |  |  |  |
| Travel History | Yes | 19 | 35 | 104 | 24 | 4 | 0.09 |  | 15 | 32 | 33 | 13 | 2 | **0.00** |
|  | No | 35 | 64 | 334 | 76 |  |  |  | 32 | 68 | 221 | 86 |  |  |
| HIV Positive | Yes | 12 | 22 | 58 | 13 | 0 | 0.10 |  | 11 | 23 | 44 | 17 | 0 | 0.31 |
|  | No | 43 | 78 | 383 | 87 |  |  |  | 36 | 77 | 212 | 83 |  |  |
| Malaria Positive | Yes | 4 | 7 | 43 | 10 | 0 | 0.81 |  | 0 | 0 | 2 | 1 | 0 | 1.00 |
|  | No | 51 | 93 | 398 | 90 |  |  |  | 47 | 100 | 254 | 99 |  |  |
| Contact with goats | Yes | 23 | 42 | 125 | 28 | 0 | **0.04** |  | 22 | 47 | 106 | 41 | 0 | 0.52 |
|  | No | 32 | 58 | 316 | 72 |  |  |  | 25 | 53 | 150 | 59 |  |  |
| Contact with cattle | Yes | 12 | 22 | 95 | 22 | 0 | 1.00 |  | 11 | 23 | 75 | 29 | 0 | 0.48 |
|  | No | 43 | 78 | 346 | 78 |  |  |  | 36 | 77 | 181 | 71 |  |  |
| Contact with sheep | Yes | 1 | 2 | 33 | 7 | 0 | 1.00 |  | 1 | 2 | 24 | 9 | 0 | 1.00 |
|  | No | 54 | 98 | 408 | 93 |  |  |  | 46 | 98 | 232 | 91 |  |  |
| Contact with swine | Yes | 0 | 0 | 1 | 0 | 0 | 0.16 |  | 0 | 0 | 1 | 0 | 0 | 0.15 |
|  | No | 55 | 100 | 440 | 100 |  |  |  | 47 | 100 | 255 | 100 |  |  |
| Contact with chicken | Yes | 32 | 58 | 180 | 41 | 0 | **0.02** |  | 30 | 64 | 148 | 58 | 0 | 0.52 |
|  | No | 23 | 42 | 261 | 59 |  |  |  | 17 | 36 | 108 | 42 |  |  |
| Contact with dogs | Yes | 12 | 22 | 73 | 17 | 0 | 0.34 |  | 10 | 21 | 38 | 15 | 0 | 0.28 |
|  | No | 43 | 78 | 368 | 83 |  |  |  | 37 | 79 | 218 | 85 |  |  |
| Contact with cats | Yes | 14 | 25 | 115 | 26 | 0 | 1.00 |  | 11 | 23 | 44 | 17 | 0 | 0.31 |
|  | No | 41 | 75 | 326 | 74 |  |  |  | 36 | 77 | 212 | 83 |  |  |
| Contact with rodents | Yes | 35 | 64 | 273 | 62 | 0 | 0.88 |  | 34 | 72 | 166 | 65 | 0 | 0.40 |
|  | No | 20 | 36 | 168 | 38 |  |  |  | 13 | 28 | 90 | 35 |  |  |
| Contact with bats | Yes | 18 | 33 | 73 | 17 | 0 | **0.01** |  | 18 | 38 | 68 | 27 | 0 | 0.11 |
|  | No | 37 | 67 | 368 | 83 |  |  |  | 29 | 62 | 188 | 73 |  |  |
| ^**)^ p values in Fisher's exact test between dengue positive and dengue negative patients | | | | | | | | | |  |  |  |  |  |
| DENV: Whole study group n= 496; pos n=55 (11%); neg n =441 (89%). Taita Taveta n= 303; pos n=47 (16%); neg n =256 (84%). | | | | | | | | | | | | | | |
